# Are we meeting local and national guidelines for physical health assessment following admission to the meadows?

**DOI:** 10.1192/bjo.2021.805

**Published:** 2021-06-18

**Authors:** Yasmin Abbasi, Ruairidh Morgan, Alice O'Docherty

**Affiliations:** Surrey and Borders Partnership NHS Foundation Trust

## Abstract

**Aims:**

We audited practice at the Meadows Inpatient Unit regarding physical health assessment, against standards set by Surrey and Borders Partnership and NICE.

**Background:**

SABP policy states that within 24 hours of admission to inpatient services, physical health assessment should be offered. It should be completed within 72 hours. Refusal should be documented.

These guidelines state that within 2 weeks of admission blood tests should be completed, and for specific individuals an ECG should be performed.

NICE guidelines state that “physical healthcare needs” should be discussed with newly admitted patients. NICE guidelines regarding physical health monitoring for individuals with psychosis or schizophrenia recommend that assessment includes “full physical examination to identify physical illness”.

NICE suggests use of antipsychotics for individuals with dementia who have severe distress, or are at risk of harming themselves or others. Those with behavioural and psychological symptoms of dementia (BPSD) should therefore be physically assessed to ensure safe use of antipsychotics may be implemented.

**Method:**

All admissions to The Meadows over seven months were audited retrospectively. The clinical notes were accessed from Systm1.

Consensus medical opinion was reached that full examination should include: GCS/level of consciousness, cardiorespiratory, abdominal and neurological examinations.

Age, gender, diagnosis and prescriptions of psychotropic medication at time of admission were recorded.

The sample included 35 patients.

**Result:**

55% of patients had a diagnosis of dementia.

63.8% of patients were prescribed antipsychotics on admission, more than other psychotropic medication. This may reflect that the most common diagnosis was dementia, commonly with associated BPSD.

97% of patients had a physical examination completed within 24 hours; most excluded neurological examination. 91% of patients had blood tests completed in two weeks, with the most commonly excluded tests being lipids and glucose. 86% of patients had an ECG in two weeks. In general, documentation of reason for not completing an examination was completed.

**Conclusion:**

We found good compliance with recommendations for physical health assessment. Areas for improvement include better assessment of neurology and more thorough blood tests.

Recommended physical health examination for new admissions is not outlined in SABP policy. We recommend the following:

GCS/level of consciousness, cardiovascular, respiratory, abdominal, and neurological examinations, and baseline observations.

ECG should be a requirement of admission. In order to facilitate this, staff need to be trained to perform ECGs.

NICE guidelines refer to HBA1c rather than glucose, which should be reflected in SABP policy.

